# New method of FACS analyzing and sorting of intact whole ovarian fragments (COPAS) after long time (24 h) cooling to 5 °C before cryopreservation

**DOI:** 10.1007/s10561-020-09898-1

**Published:** 2021-01-24

**Authors:** Wanxue Wang, Mahmoud Salama, Plamen Todorov, Dimirtry Spitkovsky, Evgenia Isachenko, Rico Bongaarts, Gohar Rahimi, Peter Mallmann, Gennady Sukhikh, Vladimir Isachenko

**Affiliations:** 1 Research Group for Reproductive Medicine and IVF-Laboratory, Department of Obstetrics and Genecology, Cologne University, Kerpener Str. 34, 50931 Cologne, Germany; 2 Feinberg School of Medicine, Oncofertility Consortium, Northwestern University, Chicago, IL 60611 USA; 3 Institute of Biology and Immunology of Reproduction (BAS), Tzarigradsko Shosse 73, 1113 Sofia, Bulgaria; 4 Institute of Neurophysiology, Cologne University, Robert-Koch-Str. 39, 50931 Cologne, Germany; 5 National Medical Research Center for Obstetrics, Gynecology and Perinatology of Ministry of Healthcare of the Russian Federation, 4 Oparina Street, Moscow, 117997 Russia; 6 Union Biometrica, Ninovesteenweg 198/16, 9320 Aalst, Belgium

**Keywords:** Flow cytometry, FACS, COPAS, Cryopreservation of ovarian tissue, Autotransplantation, Long-time cooling

## Abstract

As recently announced by the American Society for Reproductive Medicine (ASRM), human ovarian tissue cryopreservation is an established option for fertility preservation in prepubertal girls and young women undergoing gonadotoxic treatments for cancer as well as some autoimmune diseases. Proper ovarian tissue assessment before and after cryopreservation is essential to increase success rates. Ovarian fragments from 16 patients were divided into small pieces in form of cortex with medulla, and randomly divided into the following two groups. Pieces of Group 1 (*n* = 16) were frozen immediately after operation, thawed and just after thawing their quality was analyzed. Group 2 pieces (*n* = 16) after operation were cooled to 5 °C for 24 h, then frozen after 24 h pre-cooling to 5 °C, thawed and just after thawing their quality was analyzed. The effectiveness of the pre-freezing cooling of tissue was evaluated by the development and viability of follicles (Calcein-AM and Propidium Iodide) using complex object parametric analyzer and sorter machine (COPAS). Positive effect of cooling of cells to low supra-zero temperatures on their future development after re-warming has been observed. New flow cytometry- technique is suitable for the evaluation and sorting of cryopreserved whole human whole intact ovarian fragments. Long time (24 h) cooling of ovarian tissue to 5 °C before cryopreservation has a trend of a cell viability increasing.

## Introduction

As recently announced by the American Society for Reproductive Medicine (ASRM), human ovarian tissue cryopreservation is an established option for fertility preservation in prepubertal girls and young women undergoing gonadotoxic treatments for cancer as well as some autoimmune diseases. Autotransplantation of cryopreserved thawed ovarian tissue has resulted in at least 130 healthy babies worldwide with reported pregnancy and livebirth rates 33% and 25%, respectively (Fertility preservation in patients undergoing gonadotoxic therapy or gonadectomy: a committee opinion 2019).

Cryopreservation is currently known as the most suitable method for fertility preservation(Filatov et al. 2016; Vuković et al. 2019). In comparison to other established options of female fertility preservation (embryo or oocyte cryopreservation), human ovarian tissue cryopreservation and further autotransplantation have unique advantages including (1) It is the only option that can offer emergency fertility cryopreservation as it does not require prior ovarian stimulation, and thus allows immediate initiation of anticancer therapy, (2) It is the only option that can restore both endocrine and reproductive ovarian functions, and (3) It is the only option that can provide fertility cryopreservation for prepubertal girls. The major challenges facing human ovarian tissue cryopreservation and further autotransplantation are (1) how to avoid the potential risk of reintroducing malignant cells in case of cancer patients and, (2) how to increase viability and prolong lifespan of ovarian tissue transplant. To overcome such challenges, proper ovarian tissue assessment before and after cryopreservation is essential (Salama and Woodruff 2015).

For our new evaluation method, the use of the automatic COPAS measurement method can improve the efficiency of screening a large number of ovarian tissue blocks, to avoid observation through the optical microscope every time and save time for sample change step.

The aim of this study is to test the viability of cryopreserved/thawed human ovarian cortex after long-time (24 h) cooling to 5 °C before freezing by COPAS-analyzing and sorting of whole (to 0.064 mm^3^) fragments.

## Materials and methods

Except where otherwise stated, all chemicals were obtained from Sigma (Sigma Chemical Co., St. Louis, MO, USA).

The medium used for transport and dissection, the culture (basal) medium, was comprised of Leibovitz L-15 with 5% Dextran Serum Substitute (Irvine Sci., Santa Ana, CA, USA). All the patients are from 19 to 41 years old. Fresh ovarian tissue fragments were collected and kept at a temperature of 32–34 °C, and then they were transported to the laboratory within 10 min of surgery as described previously (Isachenko et al. 2012a). Using tweezers and a No. 22 scalpel, the ovarian fragments were dissected into medulla-contained small pieces: 2.0–4.0 × 2.0–4.0 × 2.0–2.5 mm). Then, the pieces were randomly divided into two groups: Group 1 (*n* = 16, average age = 30.2) were frozen in the freezing medium immediately after surgery. Group 2 pieces (*n* = 16, average age = 32.9) were cooled at 5 °C for 22–24 h in basal culture medium and were frozen the next day as described below.

### Tissue cryopreservation (freezing and thawing)

The procedure of freezing and thawing was performed as published previously (Isachenko et al. 2009a; Isachenko et al. 2012b; Isachenko et al. 2008; Isachenko et al. 2012c; Isachenko et al. 2006; Isachenko et al. 2013b; Isachenko et al. 2015).

Pieces of ovarian tissue were placed at room temperature in 20 ml freezing medium composed of basal medium supplemented with 6% dimethyl sulfoxide, 6% ethylene glycol and 0.15 M sucrose. Then pieces were put into a standard 5-ml cryo-vials (Thermo Fisher Scientific, Rochester, NY, USA) previously filled by freezing medium and frozen in a IceCube 14S freezer freezer (SyLab, Neupurkersdorf, Austria). The cryopreservation programm was as follows:(1) the starting temperature was − 6 °C; (2) samples were cooled from − 6 to − 34 °C at a rate of  − 0.3 °C/min; (3) at − 34 °C cryo-vials were plunged into liquid nitrogen. The freezing protocol for cryopreservation of this ovarian tissue included an auto-seeding step at − 6 °C.

The procedure of thawing was achieved by holding the vial for 30 s at room temperature followed by immersion in a 100 °C (boiling) water bath for 60 s, and expelling the contents of the straw into the solution for the removal of cryoprotectants. The exposure time in the boiling water was visually controlled by the presence of ice in the medium; as soon as the ice was 2 to 1 mm apex, the straw was removed from the boiling water, at which point the final temperature of the medium was between 4 and 10 °C. Within 5–10 s after thawing, the pieces from the cryo-vials were expelled into 10 ml thawing solution (basal medium containing 0.5 M sucrose) in a 100 ml specimen container (Sarstedt, Nuembrecht, Germany).

The stepwise dilution of cryoprotectants was achieved using the same principle as that used for saturation by ethylene glycol by Isachenko et al. (Isachenko et al. 2006). The container was placed on a shaker and continuously agitated at 200 osc/min for 15 min at room temperature. Stepwise rehydration of the tissue pieces for 30 min at room temperature was also performed using the same “dropping” methodology: slow addition of basal medium (see above) to the solution of sucrose with ovarian pieces. For “dropping,” we used 50 ml of basal medium in a 50-ml tube (Greiner Bio-One GmbH, Frickenhausen, Germany). The final sucrose concentration was 0.083 M, resulting in almost isotonic conditions. Finally, the pieces were washed thrice each in basal medium for 10 min and transferred for analysis.

### Complex Object Parametric Analyzer and Sorter (COPAS) analysis

For high-throughput analysis and sorting of large and fragile ovarian pieces, we use the COPAS VISION 500 (Union Biometrica, Belgium) for the analysis and dispensing of tissue fragments on the basis of size, optical density, and fluorescent parameters. The COPAS VISION 500 allows to analyze and sort particles in the diameter size range from 6–400 μm provides flexibility with regards to object size and measures several parameters: size (Time of Flight, TOF), optical density (Extinction, EXT) and the intensity of fluorescent markers. Once analyzed, objects are sorted according to user selectable criteria and then may be dispensed into stationary bulk receptacles or multi-well plates for further study. The instruments have been proven to analyze and sort (fragile) cell clusters with higher yields and recovery. To avoid damaging or changing the fragile samples, a gentle pneumatic device is located after the flow cell is used for sorting and makes the instrument suitable for handling live biological materials or sensitive chemistries. The fluid pressurization of the instrument (up to 5 Ibf/in2, psi) is also significantly lower than those of traditional flow cytometers. The Profiler option allows to view each individual object and see where fluorescence is located along the axis when passing through the flow cell. After test, data were analyzed and output using Flow Pilot-Pro software.

Ovarian fragments in different shapes may have different profiler plot in COPAS, which can be a sorter and verification style for more alive fragments for further research such as single-cell sequencing, proteomics study in ovarian tissue with different treatment groups, and improve the understanding of ovarian tissue cell types, which may improve the ovarian model establishment in future. Our study about profiler plot and imaging in COPAS Vision is the first linear description of human ovarian tissue.

**Fig. 1:**
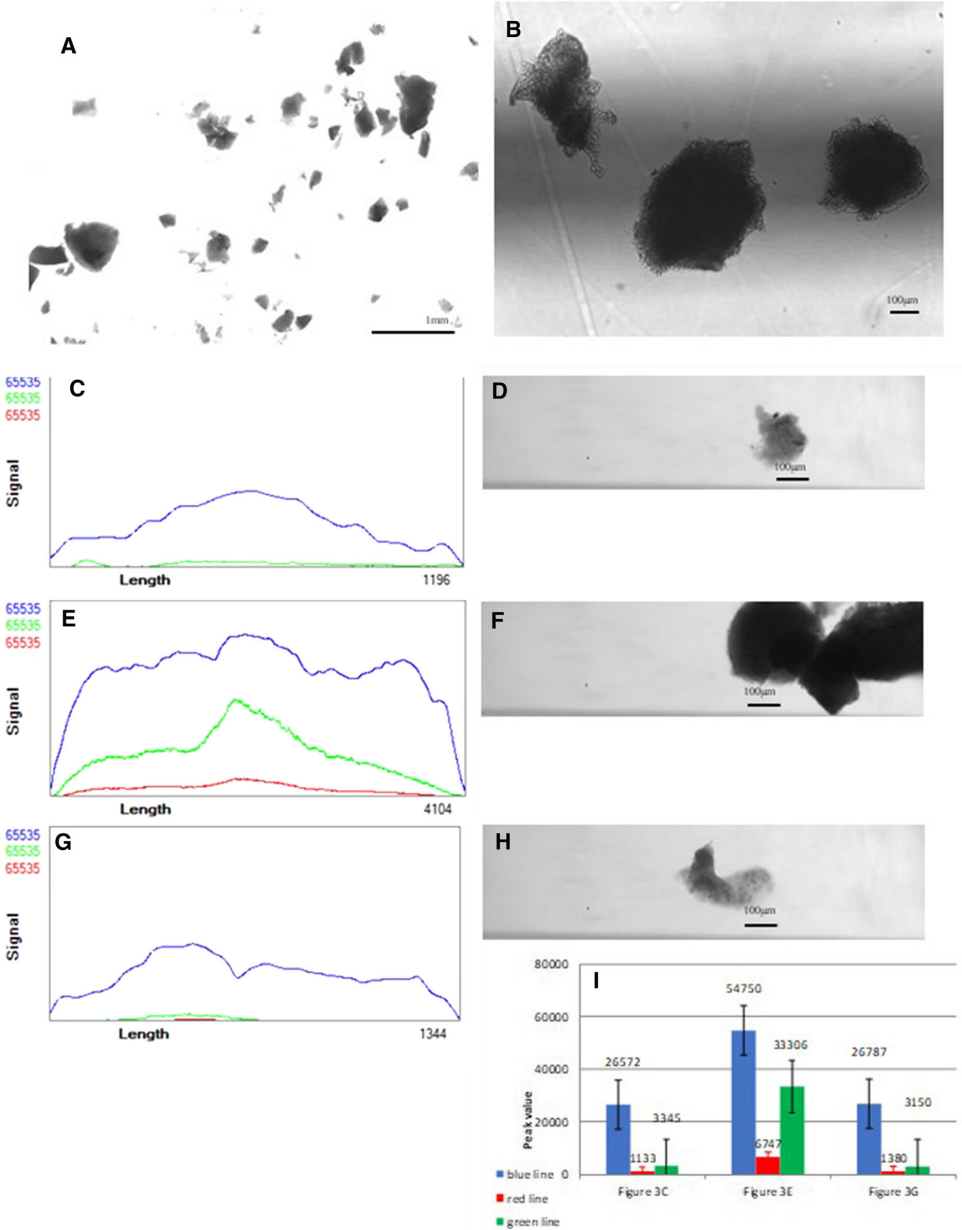
Basic characteristics of control group. **a** Microscope image of control group prepared for analysis on the COPAS Vision. **b** Microscopic image 5X of sorted fragments in control group. **c** Profiler plot of a relatively transparent and low auto-fluorescent fragment in control group. **d** Image of a relatively transparent and low autofluorescent fragment in control group. **e**. Profiler plot of a dark and medium auto-fluorescent fragment in control group. **f**. Image of a dark and medium auto-fluorescent fragment in control group. **g** Profiler plot of a smaller fragment with possible follicle in control group. **h** Image of a smaller fragment with possible follicle in control group. **i** Histogram comparing autofluorescence brightness of the different shapes of fragments in control group. (Color figure online)

**Fig. 2:**
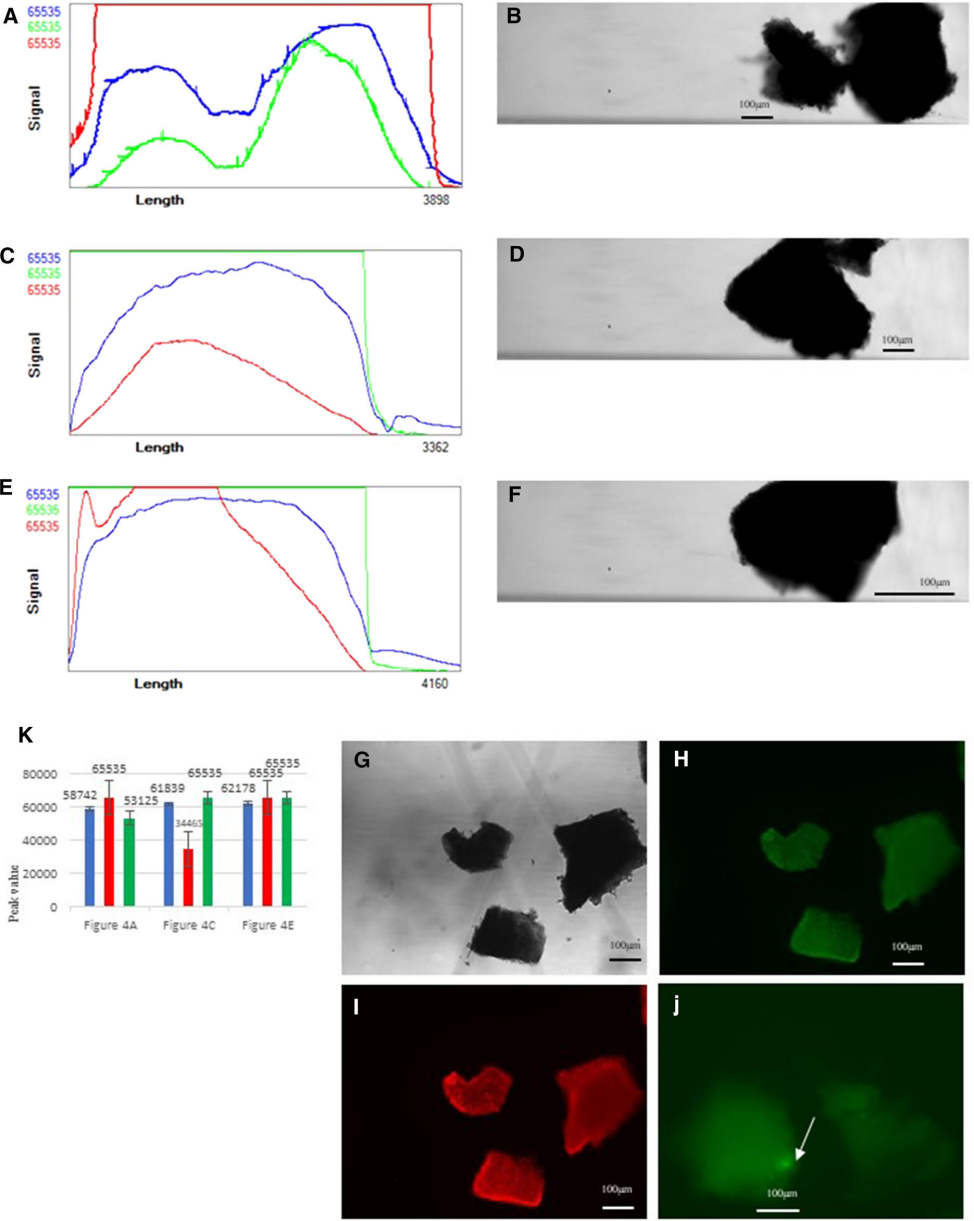
Calcein-AM and PI staining data of fragments in control group **a** Profiler plot for two adherent staining fragments. The Green line represents the Calcein staining, the Red the PI staining. Due to the high sensitivity setting of the Red PMT we saturated the red signal. The sensitivity of the PMT was reduced to 300 V . **b** Brightfield image of two adherent staining fragments in control group. **c** and **e** are two profiler plots of larger staining fragments in control group . The adaptation to the red sensitivity shows the red line as PI staining more correct. The green Calcein staining on these fragments is very high. d and f are two images of larger staining fragments in control group. **g** Microscopic images of stained fragments in brightfield. **h** Microscopic images of stained fragments in green Calcein-AM. **i** Microscopic images of stained fragments in red PI . **j** Microscopic images of stained fragments with visible follicles in green Calcein-AM. **k** Histogram comparing fluorescence brightness of the different shapes of stained fragments in control group. (Color figure online)

These ovarian tissue segments in two groups were chopped by scalpel into small pieces. Using sieving and pipetting we tried to get particles smaller than 500 microns and take microscope image for analysis on the COPAS Vision (Fig. 1a). Sample tissue fragments were diluted in medium into a 50 ml tube and analyzed. The tissue fragments varied largely in size and optical density. We also looked at the values for auto-fluorescence in the Violet and Green channel, and most objects had a relatively high level of green auto-fluorescence (Fig. 2).

Basic characteristics of control group (Fig. 1b, d, f, h) and comparing autofluorescence brightness of the different shapes of fragments in control group (Fig. 1c, e, g, i) show that the blue line and green line may higher when a fragment is larger and thicker than normal. This means that we can control the size of sorted fragments also by blue line’s value. In Fig. 1b, we can see the sorted tissue fragments show similar sizes in microscopic imaging.

**Fig. 3:**
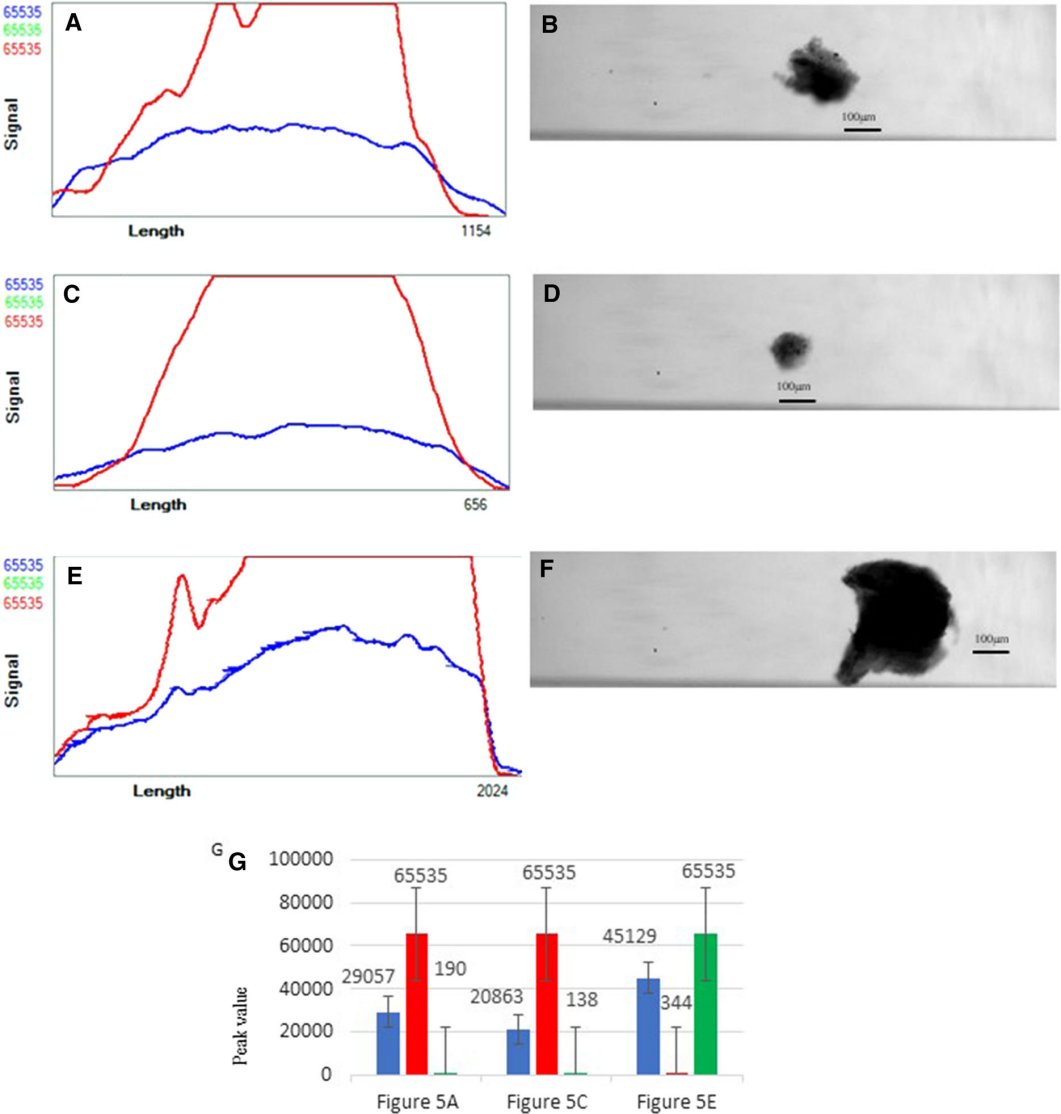
Basic characteristics of pre-cooling group. **a** Profile of a small and more transparent fragment in pre-cooling group. **b** Image of a small and more transparent fragment in pre-cooling group. **c** Profile of a possible follicle with some embedded tissue in pre-cooling group. **d** Image of a possible follicle with some embedded tissue in pre-cooling group. **e** Profile of a larger and darker fragment in pre-cooling group. **f** Image of a larger and darker fragment in pre-cooling group. **g** Histogram comparing fluorescence brightness of the different shapes of fragments in pre-cooling group. (Color figure online)

In pre-cooling group, profile and image of fragments in pre-cooling group without staining show higher red auto-fluorescence. Different shapes and structures can be observed in different trends in profiler plot of tissue in pre-cooling group (Fig. 3a–f).

The more transparent tissue has the flatter plot and the darker fragments in brightfield under microscope can acquire steeper curve of profiler plot from COPAS Vision (Figs. 1f, 3f). For the image of follicles with some embedded tissue in control group and pre-cooling group, we can notice that sometimes we can observe the follicles in it via image from this system, usually the more transparent and smaller fragments.

### Propidium iodide staining and green Calcein-AM staining

The viability of morphologically normal ovarian tissue was analyzed using live (Calcein-AM) and dead (Propidium iodide) markers. The thawed tissue pieces were transferred to PBS containing 2 μmol/l of Calcein-AM and 5 μmol/l of Propidium iodide, respectively. They were incubated with the fluorescent dyes for 20 min at 37 °C in the dark. Then the ovarian fragments were observed under COPAS and an inverted fluorescence microscope (Zeiss, Germany).

### Ethics and dissemination

The investigators declare that the study will be conducted in full compliance with Ethical supervising association in University hospital of Cologne regarding the clinical research and the principles of the Declaration of Helsinki. Study results will be disseminated through peer-reviewed journals and conferences. Ethical approval for this study has been given by Ethics Boards of University Cologne (applications 99,184 and 13–147).

### Patient and public involvement

Informed consent was obtained from all 16 patients aged between 21 and 36 (31.8 + 4.2) years. This event was attended by the principle investigators to actively seek and hear the views of the patient and public representatives. The group unanimously found that this area of research was important, found no feasibility or acceptability issues with the study and thought it likely that they would take part if asked.

### Dissemination

This study will be submitted to peer-reviewed journal and manuscript(s) will be prepared following close of the study.

## Results

In profiler plot and images for larger staining fragments in control group (Fig. 2a–f), the green line represents the Calcein staining, the red line represents the PI staining. Due to the high sensitivity setting of the Red PMT we saturated the red signal. The sensitivity of the PMT was reduced to 300 V. The adaptation to the red sensitivity shows the red line as PI staining more correct. The green Calcein staining on these fragments is very high. And we have been observing at the images in green to look for spots that had a higher concentration. Compare with COPAS imaging (Fig. 2a–f), the clarity of conventional microscopic images (Fig. 2g, h, i) is higher. But obviously, the COPAS is more timesaving for screening a large number of ovarian fragments, to avoid observation through the optical microscope and do not need change fragment samples every time. Those were visible follicles in microscopic images of green Calcein-AM stained fragments (Fig. 2j) which also can be recognized through COPAS profiler plot (Fig. 2c and e).

**Fig. 4:**
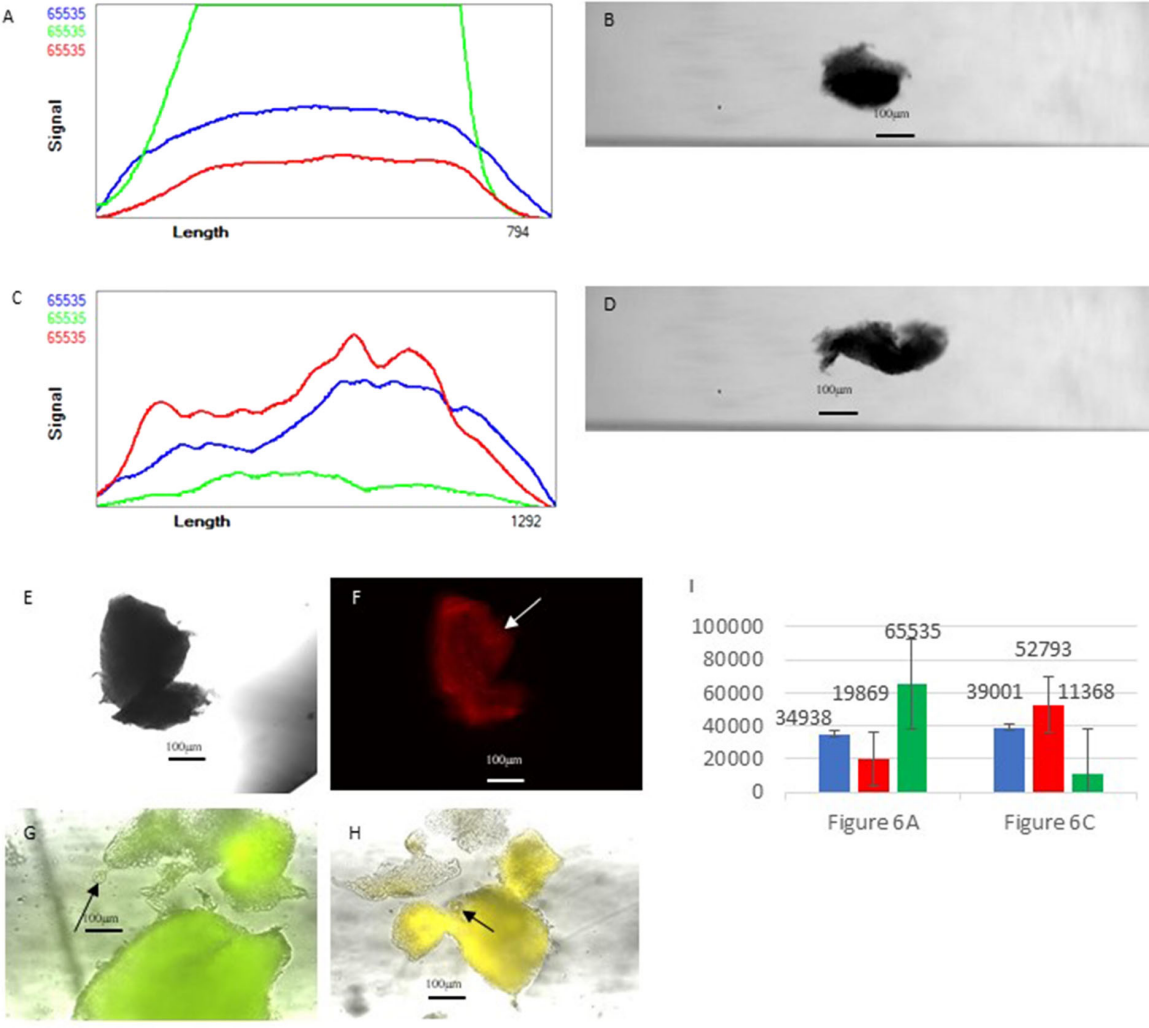
Calcein-AM and PI staining data of fragments in pre-cooling group. **a** Profile of a darker fragment in pre-cooling group with PI and high Calcein staining. **b** Image of a darker fragment in pre-cooling group with PI and high Calcein staining. **c** Profile of a transparent fragment in pre-cooling group with PI and low Calcein staining. **d** Image of a transparent fragment in pre-cooling group with PI and low Calcein staining. **e** Microscopic image 10 × of a sorted staining fragment in pre-cooling group in brightfield. **f** Microscopic image 10 × of a sorted staining fragment in pre-cooling group in red. Upper right might be an embedded follicle. **g** and** h** are microscopic images that were made from several sorted fragments in pre-cooling group to find embedded follicles. The arrows point to the follicles. (Color figure online)

In Fig. 4, there are the Calcein-AM and PI staining data of fragments in pre-cooling group. Darker fragment in pre-cooling group with PI and high Calcein staining (e.g. Fig 4a and b) shows higher Calcein-AM staining in microscopic images and in profiler plot from COPAS Flow Pilot software linear plot than the control group in average level (Fig. 4i). However, the profiles of the relatively more transparent fragments in pre-cooling group with PI and low Calcein staining (e.g. Figure 4c and d) shows higher red staining and higher red channel signal (Fig. 4 I). In Fig. 4e and f, a sorted staining fragment in pre-cooling group in red, the upper right dot (arrow pointed on Figure) might be an embedded follicle. And in Fig. 4g and h, we can find that several sorted fragments in pre-cooling group with more embedded follicles.

In general, the pre-cooling group shows higher viability. And the positive effect of cooling of cells to low supra-zero temperatures on their future development after re-warming has been observed before and is consistent with our previous research results (Isachenko et al. 2012b , 2015, 2016).

## Discussion

Analysis of single cells by flow cytometry has become a common tool for both clinicians and basic science researchers (Theunissen et al. 2017; Wagner et al. 2020; Zver et al. 2017). To help improve the grafting technique, it was investigated whether short-term xenografting of a suspension containing ovarian stromal and endothelial cells without follicles could enhance graft survival and revascularization (Dath et al. 2011). Proportions of CD34-positive cells were evaluated by flow cytometry. And also the normal type multicolor flow cytometry (FCM) is useful to evaluate the presence of viable leukemic cells in the ovarian cortex with a high specificity and a robust sensitivity (Amiot et al. 2013; Zver et al. 2014, 2015).

In our study we evaluated large particle flow cytometry as a novel automated tool for the analysis of the fragments size and follicles distribution in large tissue pieces (to 0.064mm^3^, max *d* = 400 μm). The primary objective of us is to present a new method that may reduce human-derived errors that compound the statistical limitations of small sample size by this novel method.

The COPAS has the capacity to analyze and sort large particles based upon optical density, size, and three wavelengths of fluorescence. The time of flight (TOF) parameter measures the amount of time that a particle spends within the path of the excitation laser and is directly proportional to particle size. Particles are suspended within a laminar flow stream, which aligns them vertically by their longest dimension. The larger the particle size the greater the TOF value. Because of the parabolic nature of the sample streams flow rate within the sheath stream, particle size and TOF values do not increase linearly. Reflective of this fact was the observation that the most accurate line fit of particle size versus mean TOF value was achieved using a second order polynomial regression model. Microspheres from 6 to 400 μm were measured with results within 5% of the manufacturer’s specifications. We also found that the fluidics and data acquisition components of the COPAS consistently produced sample recoveries greater than 94% for all microsphere sizes tested. These data show that the COPAS flow cytometer has the precision and accuracy required to detect, discriminate, and sample particles within the range of sizes typically observed for human ovarian tissue.

We provide the first data demonstrating that large particle flow cytometry is applicable to the study of intact ovarian fragments. As an evaluation method for clinicians, the COPAS can accurately determine live and died ovarian fragments by staining with Calcein-AM and PI. This is an important feature because the success of ovarian autotransplantation has been shown to partially depend upon ovarian pieces survival (Isachenko et al. 2013a; Isachenko et al. 2009b). As a tool for ovary researchers, the COPAS provides the ability to select specific ovarian pieces based upon specific user defined criteria (e.g. size, or biological markers). Expanding the capabilities of the COPAS will be accomplished by utilization of additional fluorescent probes of ovarian metabolism and function.

At present, the storage of organs by low temperatures is a widely used technology (Southard and Belzer 1995; Wang et al. 2002). An absence of negative effect and even positive effect of cooling of cells to low supra-zero temperatures on their future development after re-warming has been observed before and is not new (Isachenko et al. 2020; Lopez-Urueña et al. 2016; Martins et al. 2018; Wasilewska et al. 2016). It was reported a good survival of bovine trophoblastic fragments that had been subjected to cooling at 4 °C for 48 h. And the survival/formation of vesicles in these fragments was not different from that of the untreated controls (98% and 98%, respectively) (Isachenko et al. 1993).

The aim of investigations performed by Wood et al. was to study the influence of long-term hypothermic storage of whole domestic cat ovary for 48 h at 4 °C on follicle-oocyte atresia and temporal taphonomy (Wood et al. 1997). It was found that the highest (but statistically insignificant) degeneration rate of follicles occurred at 48 h, with inhibition of taphonomy. Our data support these results.

It was shown that cooling of the warm-blooded rats stimulates mitosis indirectly in cells capable of division. It is happened because this treatment stimulates directly the mitotic activity in mouse and human cells cultured and adapted to the cold in vitro (Fiedler et al. 2006). It was established by in situ hybridization analysis of hypothalamic tissue that cold exposure causes a two-fold increase in the total number of neurons expressing thyrotrophin-releasing hormone mRNA in the paraventricular nucleus. It was found that hypothermic storage of rat ovary at 4 °C for 24 h did not disrupt ovarian function (Yin et al. 2003).

The exposure of human ovarian tissue to low positive temperatures of up to 26 h does not.

inhibit the development of follicles during subsequent in vitro culture. In compaison with the.

untreated controls, the number of developing primordial follicles in tissues of all treatment groups was significantly decreased (Isachenko et al. 2009a).

The aim of the performed on ovarian tissue experiemnts was to study the intensiveness of neo-vascularisation and follicular development in ovarian tissue after 24 h cooling to 5 °C before cryopreservation. It was established that the long-time cooling before is beneficial for cryopreservation of human ovarian tissue (Isachenko et al. 2012b).

The aim of the following study was to test the intensiveness of the phosphatidylserine translocation immediately after thawing and after 45 d xenografting of human ovarian tissue, which was either frozen just after operative removal from patient or cooled before cryopreservation to 5 °C for 24 h and then frozen (Isachenko et al. 2015). Phosphatidylserine is a phospholipid component of membrane which plays a key role in cell cycle signaling, specifically in relationship to necrosis and apoptosis. When a cell affected by some negative factors, phosphatidylserine is no longer restricted to the intracellular side of membrane and translocated to the extracellular surface of the cell. This is they act as a signal for macrophages to engulf the cells (Verhoven et al. 1995). At least five negative effects observed during cells cryopreservation: hypoxia, increasing of intracellular Ca2 + , osmotic disruption of cellular membranes, generation of reactive oxygen species (ROS) and lipid peroxidation(Cacciottola et al. 2018; Damous et al. 2014; Isachenko et al. 2016; Talevi et al. 2013). Each from these factors can lead to translocation of phosphatidylserine. It can be concluded that cooling of ovarian tissue to 5 °C for 24 h before cryopreservation decreased translocation of phosphatidylserine that evidences about increases the viability of the cells in the tissue after thawing.

## Data Availability

The data used to support the findings of this study are included in the article.
